# Recent advances in understanding the cellular roles of GSK-3

**DOI:** 10.12688/f1000research.10557.1

**Published:** 2017-02-20

**Authors:** Kevin W. Cormier, James R. Woodgett

**Affiliations:** 1 Lunenfeld-Tanenbaum Research Institute, Sinai Health System, 600 University Avenue, Toronto, ON, M5G 1X5, Canada

**Keywords:** GSK-3, glycogen synthase kinase-3, GSK-3 isoform, GSK-3 biology, GSK-3 post-translational modification

## Abstract

Glycogen synthase kinase-3 (GSK-3) is a ubiquitously expressed protein kinase that sits at the nexus of multiple signaling pathways. Its deep integration into cellular control circuits is consummate to its implication in diseases ranging from mood disorders to diabetes to neurodegenerative diseases and cancers. The selectivity and insulation of such a promiscuous kinase from unwanted crosstalk between pathways, while orchestrating a multifaceted response to cellular stimuli, offer key insights into more general mechanisms of cell regulation. Here, we review recent advances that have contributed to the understanding of GSK-3 and its role in driving appreciation of intracellular signal coordination.

## Introduction

Initially discovered as a contributor to regulating the rate-limiting step of glycogen synthesis via phosphorylation and deactivation of glycogen synthase ^1^, glycogen synthase kinase-3 (GSK-3) has since been associated with a variety of other signaling pathways. These include cyclic adenosine monophosphate (cAMP) signaling, Wnt, Hedgehog, Notch, transforming growth factor-beta (TGF-β), nuclear factor of activated T cells (NF-AT), and agonists that act via stimulation of phosphatidylinositol 3-kinase (PI3K) ^2– 4^. To date, over 40 GSK-3 substrates have been identified ^5^, and over 500 others as potential candidates remain to be validated ^6^. With such a broad range of pathway involvement and substrate interactions, it is unsurprising that GSK-3 participates in the regulation of multiple cellular functions, including metabolism, cell motility, apoptosis, cell differentiation, proliferation, and embryonic development, and that its dysregulation is implicated in various diseases, including mood disorders, diabetes, Alzheimer’s disease (AD), and several forms of cancer ^7, 8^. Depending on the type of tumour tissue, GSK-3 can act as either a tumour promoter or suppressor ^9^. The multiplicity of actions of this protein kinase, coupled to its insinuation into diverse cellular regulatory processes, creates challenges in understanding its true functions and provides deeper insights into the interconnectivity of cellular control circuitry. While in no way attempting to be exhaustive, we will offer some highlights from the last three years of research on GSK-3 that have provided new appreciation as well as avenues for future research.

GSK-3 is a ubiquitously expressed serine/threonine kinase that exists as two isoforms (GSK-3α and GSK-3β) encoded by two distinct genes ^10^. The two proteins share 98% identity in their kinase domain, but GSK-3α has a glycine-rich N-terminal extension that accounts for its larger mass (51 kDa compared with 47 kDa for GSK-3β). There are also differences in their C-termini, with only 36% identity in the last 76 residues. It is distinguished among kinases for preferring substrates that are primed by a prior phosphorylation event C-terminal to the GSK-3 phosphorylation site ^11^. The priming phospho-serine or threonine binds within a positively charged pocket (created by R98, R180 and K205 in GSK-3β) such that a serine or threonine 3-4 residues N-terminal to the substrate’s priming residue is directed to the GSK-3 active catalytic site. GSK-3 also has the rare characteristic of being active under basal conditions. It can instead be inhibited by phosphorylation by upstream kinases (S21 for GSK-3α and S9 for GSK-3β) ^12^, the phosphorylated serine acting as a pseudo-substrate at the priming phosphorylation binding pocket resulting in competitive inhibition ^13, 14^.

## Recent advances in GSK-3 biology

### Wnt/STOP

In addition to regulation by phosphorylation, GSK-3 activity is modulated through association with scaffolding proteins, promoting its association with some substrates and allowing insulation from others. A significant challenge in the field of cell signaling has been to understand how a single protein kinase or signaling component may participate in the control of multiple, distinct pathways. Together with dynamic considerations, such as subcellular localization, concentration, activity, and modification state ^15^, scaffolding proteins ^16^ form part of the answer. The best characterized interaction between GSK-3 and a scaffold is its association with Axin. Less than 10% of the total GSK-3 molecules (α or β) in a cell is associated with Axin, but only GSK-3 bound to Axin is capable of phosphorylating β-catenin as part of the canonical Wnt signaling pathway ^17^. Through this sequestration, Axin consigns a portion of the GSK-3 cellular pool to Wnt-related signaling. In the absence of Wnt ligands, a destruction complex is formed comprising Axin, adenomatosis polyposis coli (APC), casein kinase-1α (CK1α), GSK-3, β-transducin repeat containing protein 1 (β-TrCP) and β-catenin. This complex maintains low levels of cytosolic β-catenin. After a priming phosphorylation by CK1α, GSK-3 further phosphorylates β-catenin allowing it to be recognized and polyubiquitinated by the E3 ligase, β-TrCP, and fating it for degradation by the 26S proteasome ^18, 19^. Upon stimulation by Wnt, the co-receptors Frizzled (Fzd) and low-density receptor-related protein (LRP5/6) cluster together with another scaffolding protein, Disheveled (Dvl). Activated Dvl binds to Axin, bringing GSK-3 and CK1 into proximity with LRP5/6. A recent structural and kinetic study of LRP6 peptide with a Ser/Pro-rich sequence showed not only that Axin-bound GSK-3 phosphorylates LRP5/6 but also that phospho-LRP5/6 motifs act as direct inhibitors of GSK-3 ^20^. GSK-3 thus fashions its own inhibitor by phosphorylating LRP5/6. Axin is also phosphorylated by GSK-3 and, in this state, adopts an “open” conformation that associates with β-catenin and LRP5/6. When GSK-3 is inhibited, the equilibrium shifts towards dephosphorylation of Axin by protein phosphatase-1 (PP1) and to a “closed” conformation that diminishes its association with β-catenin and LRP5/6 ^21^. β-catenin thereby accumulates in the cytoplasm and translocates to the nucleus where, associated with TCF/LEF (T-cell factor/lymphoid enhancer binding factor) DNA binding proteins, it regulates gene expression. Dephosphorylated Axin auto-inhibits through an intermolecular interaction of its β-catenin binding domain (BCD) and its DIX domain, and high concentrations of β-catenin may win out the competitive inhibition, thus triggering reformation of the complex and regulation of β-catenin levels ^21^. These studies of LRP5/6 and Axin provide insight into the mechanism of signaling regulation and draw attention to the role of conformational changes and allosteric interactions that are affected by phosphorylation.

Although the above schema describes the canonical Wnt/β-catenin signaling pathway, there has been recent focus on β-catenin-independent Wnt signaling, as reviewed by Acebron and Niehrs ^22^. LRP5/6-bound Wnt does not simply feed into canonical Wnt signaling but can branch off to regulate other pathways. Since phosphorylation by GSK-3 can prime many proteins, in addition to β-catenin, for E3 ubiquitin ligase recognition and eventual proteasomal degradation, suppression of GSK-3 activity leads to stabilization of these proteins. This process of Wnt-dependent stabilization of proteins has been referred to as Wnt/STOP and peaks in the G _2_/M phase of the cell cycle ^23^. Wnt/STOP signaling is postulated to slow degradation of proteins as cells prepare to divide. The Niehrs lab recently showed that Wnt/STOP is independent of β-catenin by studying Wnt signaling in sperm where effects of transcription can be excluded ^24^. In another branch of Wnt/LRP5/6 signaling, referred to as Wnt/TOR, tuberous sclerosis complex 2 (TSC2) phosphorylation by GSK-3 is inhibited such that TORC1 is no longer repressed and protein synthesis is promoted ^25^. Together, these three branches of Wnt/LRP5/6 signaling control various aspects of the cellular proteome by promoting gene expression (Wnt/β-catenin) and protein synthesis (Wnt/TOR) and by decreasing protein degradation (Wnt/STOP). What is less clear is whether these pathways are tied to the theory that GSK-3 can be sequestered into multivesicular bodies during Wnt signaling ^26^. Chronic activation of Wnt components is reported to lead to association of the Axin complex with the endocytotic LRP6 signalosome, giving rise to multivesicular bodies that physically separate GSK-3 from the cytosol by two lipid bilayers. However, there are a number of caveats of the original experimental evidence, as outlined by Metcalfe and Bienz ^27^.

A defined subset of proteins may be stabilized by Wnt/STOP in a concerted fashion to set the stage for mitosis. Identification of those proteins stabilized in this manner may uncover new players in cell cycle control and processing. It is also possible that there are distinct subsets of regulatory protein complexes within the Wnt/STOP response that are dedicated to particular compartments.

### GSK-3 regulation by post-translational modifications

Besides exploring the regulation of GSK-3 through association with scaffolding proteins and phosphorylation at S9/21, recent findings have explored GSK-3 regulation by other post-translational modifications. A distinct phosphorylation site occurs at S389 on GSK-3β (GSK-3α lacks this residue) ^28^. With a mouse model in which S389 was mutated to alanine to prevent phosphorylation, it was shown that this modification restrains GSK-3 activity independently of S9 phosphorylation. GSK-3β is phosphorylated at this site by p38 mitogen-activated protein kinase (MAPK). Phosphorylation of GSK-3β at S389 occurs predominantly in the thymus ^29^ and is induced in response to DNA double-strand breaks. Inactivation of GSK-3β leads to stabilization of induced myeloid leukemia cell differentiation protein (Mcl-1), which is important for promotion of cell fitness and protection against apoptosis.

ADP-ribosylation occurs via addition of ADP-ribose from the co-factor β-NAD ^+^ to a protein substrate. Like phosphorylation, this reaction is reversible through the action of ADP-ribosyltransferases in the forward direction and ADP-ribosylhydrolases in the reverse direction. Recently, ARTD10 was identified as catalyzing mono-ADP-ribosylation of GSK-3β. This results in decreased *in vitro* kinase activity of GSK-3β. This inhibition has also been observed in U2OS cells that co-express GFP-ARTD10 and GSK-3β ^30^. Complementing this finding was the discovery that MacroD2 acts as a mono-ADP-ribosylhydrolase that removes the ADP-ribose group from GSK-3β, thus restoring its activity both *in vitro* and in cells ^31^.

GSK-3β is also citrullinated within its N-terminal domain by protein arginine deiminase 4 (PAD4) in a reaction in which arginine residues are converted to (uncharged) citrulline residues. This modification was observed to promote nuclear accumulation of GSK-3β ^32^. In a distinct study, ubiquitination of GSK-3 at lysine 63 by the E3 ligase TNF receptor-associated factor 6 (TRAF6) was shown to be essential for incorporation of GSK-3 into a Toll-like receptor 3-assembled multiprotein complex that then activated an ERK and p38 MAPK immune response via pro-inflammatory cytokine production ^33^. Only GSK-3β (not GSK-3α) was involved in TLR3-mediated pro-inflammatory cytokine production. This was determined by inhibition of inflammatory cytokines—including interleukin-6 (IL-6), tumour necrosis factor-alpha (TNF-α) and IL-10—in GSK-3β selectively silenced bone marrow-derived macrophages.

### Non-redundancy of GSK-3 isoforms

Despite their high degree of structural similarity, the two isoforms of GSK-3 are not functionally equivalent. Homozygous inactivation of GSK-3β is embryonically lethal for mice because of massive liver degeneration or cardiac patterning defects, and GSK-3α is unable to rescue this phenotype ^34^. GSK3-α null mice, on the other hand, are viable. The research literature has also tended to focus on GSK-3β and has largely neglected GSK-3α.

Two recent studies, however, have looked specifically at GSK-3α. In the first, GSK-3α global knockout mice were observed to have a shorter lifespan than their wild-type littermates. This led to the discovery that GSK-3α is a suppressor of ageing, retarding age-related pathologies in the heart, liver, small intestine, bones and joints ^35^. Although this study did not focus on the molecular mechanisms by which GSK-3α mediated these effects, two pathways that involve autophagy were implicated. Activation of protein kinase B (Akt/PKB) via the insulin/insulin-like growth factor 1 (IGF-1) signaling pathway failed to impair autophagy in GSK-3α knockout mice. Moreover, loss of inhibition of the mechanistic target of rapamycin (mTOR) pathway was critical to the ageing phenotypes. GSK-3 inhibits mTOR complex 1 (mTORC1) via interaction with TSC2, and unrestrained activity of mTORC1 in GSK-3α knockout mice led to significant inhibition of autophagy, impairment of which was proposed to promote ageing.

A second study focusing on GSK-3α showed that this isoform plays a role in atherosclerosis, a disease of the medium and large arteries in which arterial walls are inflamed and accumulate lipids. In this study, GSK-3α knockout mice were crossed with low-density lipoprotein receptor-deficient (Ldlr ^−/−^) mice and effects of GSK-3α deficiency on high-fat diet-induced atherosclerosis were examined ^36^. When placed on a high-fat diet, both heterozygous and homozygous GSK-3α knockout mice developed significantly smaller atherosclerotic lesions and had significantly less hepatic lipid accumulation compared with the *Ldlr* ^−/−^, *Gsk3a ^+/+^* control mice. *In vitro* treatment of thioglycolate-elicited peritoneal macrophages with glucosamine or tunicamycin revealed a fourfold increase in IL-10 expression in GSK-3α-deficient macrophages compared with animals with wild-type GSK-3α. Elevated levels of IL-10 were also detected in high-fat diet-fed mice that lacked GSK-3α. These data point to possible roles of GSK-3α in regulation of pro-inflammatory and anti-inflammatory responses, as has also been suggested for GSK-3β in other systems ^37^.

### Inhibitor development

Although there has been a slowly growing literature that distinguishes GSK-3α from GSK-3β, functional redundancy of the two isoforms may turn out to be an effective means of regulating total GSK-3 activity should an isoform-specific inhibitor be developed. All currently available small-molecule inhibitors are equipotent towards both isoforms. Moreover, the pro-oncogenic effects of GSK-3 inhibition on β-catenin stabilization require greater than 75% inhibition, and no impact is observed if only one isoform is inactivated. This selective therapeutic value is further supported by the fact that birds that have no GSK-3α have significantly lower levels of tau phosphorylation, one of the pathologies associated with AD ^38^. However, the structural similarity of isoforms, especially in proximity to the ATP binding site (the common target of most inhibitors), has stymied development of isoform-specific inhibitors to date.

A possible alternative approach takes advantage of inherent specificity of substrates for their respective kinases. Substrate-competitive inhibitors provide a promising route to selectivity that also yields more moderate levels of inhibition, which may be desirable in the treatment of chronic diseases where target ablation is deleterious ^39^. This approach led Eldar-Finkelman *et al*. to look at developing a GSK-3-specific inhibitor from a substrate peptide. Building on previous work in which they developed a peptide lead compound derived from heat shock factor-1 (HSF-1) ^40^, they improved on the compound such that it acts as both a substrate and an inhibitor. By retaining the phosphorylation site, the second-generation compound (termed L807) is first phosphorylated and then becomes inhibitory ^41^. By molecular dynamic simulation, these authors suggest that the mechanism of inhibition relies on phosphorylation changing the conformation of L807 and shifting it within the substrate binding trough to lock down on the peptide. When dually phosphorylated, the L807 peptide forms a stable hydrophobic center within itself and the two phosphorylated serine residues bind to the positively charged priming site cavity on GSK-3, forming hydrophobic contacts with the substrate binding pocket. Cell permeability was achieved by addition of a C14 fatty acid to the N-terminal end of the peptide to yield L807mts. As a bonus, this N-terminal conjugation appears to enhance pharmacological properties, as compound degradation is typically the weakness of peptide-based inhibitors. Rather, L807mts is stable, can penetrate the blood-brain barrier, and is non-toxic in mice at effective doses. Mice treated with L807mts exhibited improved cognitive and social behaviours in an AD model. The authors postulate that L807mts acts to increase autophagic flux, thereby clearing β-amyloid plaques present in the disease model. What is most exciting about this work is that it opens up a new way of thinking about kinase inhibitor development in that the strategy relies on the kinase to create its own inhibitor that is then resistant to dissociation by virtue of being phosphorylated.

We also note that the clinical development of GSK-3 inhibitors has not been rosy as a series of highly selective and potent small molecules have not made it past pre-clinical assessment. One exception is the non-ATP competitive inhibitor tideglusib or NP03112. This molecule reached phase II clinical trials for the treatment of AD ^42^. Although the results of this trial suggested that the drug was safe over the 26-week course of the study, no clinical benefits were observed for patients with mild to moderate AD. There were promising results, however, in a low-dose group suggesting that its pharmacological action should be further examined to find optimal conditions for inhibition, especially given that no other drugs have yet proven successful in halting progression of this disease.

## Future considerations

The enduring view of signal transduction that is still espoused in textbooks and product catalogs is that signaling is largely linear with some arrows linking feedback mechanisms and crosstalk. As numerous proteomic studies have amply demonstrated, proteins are not organized in such simplistic cascades and instead are clustered into macromolecular complexes, often tasked with subspecialized functions. This view of signaling helps to explain the exquisite level of tuned responses within cells as well as overall coordination of responses. Yet our approaches to tampering with signaling molecules for therapeutic use still suppose that these targets are arranged in discrete pathways. Moreover, our experimental tools are largely agnostic to the intricate decoration of signaling hubs and clusters and take an indiscriminate approach to blocking their targets—treating all of the target molecules identically, regardless of their disposition within subcellular complexes. This is true of small interfering RNA (siRNA), gene knockouts, drugs and CRISPR/CRISPR-associated protein-9 nuclease (Cas9). This may also explain the low threshold for adverse side effects, especially in chronic diseases where the targets are not wildly active and play essential roles in multiple, unaffected tissues. If this is true, it also represents a largely untapped opportunity for increased selectivity and precision in therapeutic targeting. In the case of GSK-3, for example, blocking its association with specific scaffolding molecules or regulatory elements may allow modulation of a problematic signaling mode while leaving its functions in other systems unharmed. Greater appreciation of the subcellular geography and microdomains of protein assemblies should also lead to better predictive modeling of normal and disease conditions and consign the simplistic, two-dimensional regulatory circuits to historical rather than teaching books.
